# Glutathione S-transferase gene GSTM1, gene-gene interaction, and gastric cancer susceptibility: evidence from an updated meta-analysis

**DOI:** 10.1186/s12935-014-0127-3

**Published:** 2014-11-30

**Authors:** Xianjun Lao, Qiliu Peng, Yu Lu, Shan Li, Xue Qin, Zhiping Chen, Junqiang Chen

**Affiliations:** Department of Clinical Laboratory, First Affiliated Hospital of Guangxi Medical University, Nanning, Guangxi Zhuang Autonomous Region China; Department of Occupational Health and Environmental Health, School of Public Health at Guangxi Medical University, Nanning, Guangxi Zhuang Autonomous Region China; Department of Gastrointestinal Surgery, First Affiliated Hospital of Guangxi Medical University, Nanning, Guangxi Zhuang Autonomous Region China

**Keywords:** Gastric cancer, Genetic polymorphism, Glutathione S-transferase M1, Gene-gene interaction, Meta-analysis

## Abstract

**Background:**

The null genotype of GSTM1 have been implicated in gastric cancer risk, but numerous individual studies showed mixed, or even conflicting results. Thus, a meta-analysis was performed.

**Results:**

We identified 54 individual studies involving 9,322 cases and 15,118 controls through computer-based searches of PubMed, Embase, and Cochrane Library. It was found that the null genotype of GSTM1 was associated with an increased gastric cancer risk (OR = 1.207, 95% CI: 1.106-1.317, P < 0.001), under the random-effects model (I^2^ : 49.9%, P_Q_ <0.001). From stratification analyses for ethnicity, alcohol drinking, *Helicobacter pylori* infection, an effect modification of gastric cancer risk was found in the subgroups of ethnicity, smoking status, *Helicobacter pylori* infection, whereas null result was found in the subgroups of alcohol drinking. We also undertook gene-gene interaction analysis between GSTM1 and GSTT1 genes for gastric cancer risk, and the results indicated that the dual null genotypes of GSTM1 and GSTT1 might elevate the risk of gastric cancer (OR = 1.505, 95% CI: 1.165-1.944, P = 002).

**Conclusions:**

This meta-analysis suggests that the null genotype of GSTM1 may be a important genetic risk factor for gastric cancer development.

## Background

The incidence and mortality of gastric cancer (GC) has been substantially falling over the last few decades in most parts of the world [1], however, gastric cancer was still the second most common cancer worldwide (989,600 new cancer cases) and also the second most common cause of cancer mortality (738,000 deaths) in 2008 [2]. As a long, complicated and multi-factorial process, gastral carcinogenesis is still not fully understood. Several suspected environmental risk factors for the development of gastric cancer are dietary habits, including high consumption of salty food and low consumption of fresh fruits and vegetables, cigarette smoking, and alcohol consumption, as well as *Helicobacter pylori* infection [3-5]. In addition to these, genetic factors also play an important role in gastric cancer aetiology, demonstrated by the fact that a large proportion of individuals with the known environmental risk factors never develop gastric cancer while many gastric cancer cases develop among individuals without these known environmental risk factors. Therefore, investigation of the responsible genetic polymorphism which may increase host susceptibility to gastric cancer is equally important to the identification of environmental risks for a better understanding of interindividual variation in response to carcinogen exposures and cancer susceptibility [6,7].

Glutathione-s-transferases (GSTs) are one of the most important supergene family of phase II isoenzymes known to catalyze the detoxification of reactive electrophilic compounds, such as carcinogens, therapeutic drugs, environmental toxins, and products of oxidative stress, chiefly by conjugation with soluble glutathione [8]. In addition, GSTs are able to modulate the induction of other enzymes and proteins which are important in cellular functions, such as DNA repair, and are therefore important in maintaining genomic integrity [9]. In this respect, the GST enzymes could potentially play a central role in carcinogenesis. In humans, GSTs are divided by electrophoresis into at least four major classes, namely *Alpha, Mu, Pi, Theta* [10]. GSTM1 and GSTT1 are the genes that encoding the *Mu* class and the *Theta* class of GSTs, respectively. The GSTM1 gene is located on chromosome 1p13.3 and contains 10 exons, and the GSTT1 gene is mapped to chromosome 22q11.23 and contains six exons. The common variants of GSTM1 and GSTT1 genes is the homozygous deletion (null genotype), which has been reported to causes the loss of enzymic activity and might higher the risk of various cancers. A recent meta-analysis [11] has suggested that an increase in gastric cancer risk was associated with GSTT1 deficiency. However, a review of genetic susceptibility and gastric cancer risk reported that the results of case–control studies detailing associations between the GSTM1 gene and gastric cancer risk are controversial [12]. Since Strange et al. firstly published the study showing an association between GSTM1 null genotype and a possible excess risk of developing gastric cancer in 1991 [13]. Subsequently, numerous researchers have consecutively reported on the same issue in various populations, but with mixed, or even conflicting results [14-65]. One of major problems with the published studies is that many of them included relatively small sample size. Furthermore, because the GSTM1 null genotype is regarded as a potential contributor to gastric cancer risk by influencing detoxification of activated environmental carcinogens and by interaction with unfavourable GSTT1 polymorphism, the possible modifying effects of GSTM1 status on the relationship between smoking status, alcohol drinking,*Helicobacter pylori* infection,GSTT1 polymorphism and gastric cancer risk is of great interest, even though not often investigated.

To obtain more precise estimate for the association between GSTM1 polymorphism and gastric cancer risk, we conducted a quantitative meta-analysis of all available studies published until August 15, 2014. In addition, we performed subgroup analysis stratified by smoking status, alcohol drinking and *Helicobacter pylori* infection to explore the possible effects of the interactions between GSTM1 genotype and above environmental risk factors, and gene–gene interaction analysis between GSTM1 and GSTT1 genotype with respect to gastric cancer risk.

## Materials and methods

### Data sources and search

We conducted a comprehensive database searching for PubMed, Embase, and Cochrane Library through August 15, 2014 for relevant studies that estimated the association between GSTM1 polymorphism and risk of gastric cancer using the following search terms: (1) gastric cancer, gastric carcinoma, gastric adenocarcinoma, stomach neoplasm, stomach cancer, GC; (2) Glutathione-s-transferases, GSTs, GST mu, GSTM, GSTM1, GST1; (3) polymorphism, SNP, variant, mutation, genetic polymorphism. The scope of the computerized literature search was also expanded on the basis of the reference lists of eligible articles. There was no restriction on language.

### Eligibility criteria and study selection

We first performed an initial screening of titles or abstracts to find potentially appropriate articles. A second screening was based on full-text review to identify those containing useful data on the topic of interest for inclusion in the meta-analysis. Studies were considered eligible if they met the following criteria: (1) publications assessed the relationship between GSTM1 status and gastric cancer; (2) used a cohort or case–control studies design; (3) had an appropriate description of GSTM1 status in cases and controls; (4) repored an odds ratio (OR) with 95% confidence interval (CI) or other available data for calculating OR (95% CI). Furthermore, when data from a single unique study population was republished by the same author or written in English, only the most recent article or largest report was considered. When a study reported the results on different subpopulations, we treated them as separate studies in the meta-analysis.

### Data extraction

Each article was extracted by two independent researchers (X Lao and Q Peng), who are blinded with respect to the authors, institutions and journals, using a structured sheet and entering into a database. The following data were extracted: first author, year of publication, country, ethnicity of study populations (categorized as Asian, Caucasian, and Negroid), number of cases and controls, gastric cancer diagnosis method, source of control selection, matching criteria between cases and controls, genotyping method, exposures of smoking, alcohol consumption, *Helicobacter pylori* infection or GSTT1 genetic polymorphism in cases and controls, GSTM1 status in cases and controls. If there were any discrepancy between these two investigators, a discussion would be carried out to make an ultimate decision through the third investigator (S Li).

### Data synthesis and statistical analysis

The strength of the association between the GSTM1 polymorphism and gastric cancer risk was measured by the odds ratio (OR) with 95% confidence interval (CI). The significance of the pooled OR was determined by Z test and a P value of less than 0.05 was considered significant. Then, we examined the associations between null genotype of GSTM1 and gasreic cancer risk on the genetic comparison model (null genotype vs. present genotype).

In carrying out the meta-analysis, two models for dichotomous outcomes were conducted: the random-effects model and the fixed-effects model. The random-effects model, using the DerSimonian-Laird method [66], was conducted to pool the results when heterogeneity between studies existed on the basis of Q-test P-value which was less than 0.1 [67]. The fixed-effects model, using the Mantel-Haenszel method [68], was utilized to pool the results if the Q-test P value was more than 0.1. Besides, the I^2^ statistic was calculated to assess the between-study heterogeneity, and heterogeneity was deemed as apparent when the I^2^ statistic value was greater than 50%. Furthermore, several subgroup meta-analyses were performed in an attempt to assessed the association between the GSTM1 null genotype and gastric cancer risk based on the ethnicity, smoking status, alcohol drinking and *Helicobacter pylori* infection. For these purposes, we stratified subjects (both GSTM1 present and null genotypes) according to ethnicity (categorized as Asians, Caucasians and Negroids ), smoking status (non/ever smokers); alcohol drinking (non/ever drinkers); *Helicobacter pylori* infection (negative/positive infection). In order to evaluate the presence of a biological interaction between GSTM1 and GSTT1 polymorphisms, additional gene–gene interaction analysis were performed by using the individuals with present genotypes for both genes as reference groups, as suggested by Botto and Khoury [69].

To validate the credibility of outcomes in this meta-analysis, a sensitivity analysis was performed by sequential omission of individual studies. Publication bias was investigated using a funnel plot, in which the standard error of logor of each study was plotted against its logor. An asymmetric plot suggested the existence of possible publication bias. In addition, funnel-plot asymmetry was formally assessed by the method of Egger’s linear regression test [70]. If publication bias existed, the Duval and Tweedie non-parametric “trim and fill” method was used to adjust for it [71]. All analyses were performed using Stata software, version 12.0 (Stata Corp, College Station, TX). All P values were two-sided. To ensure the reliability and the accuracy of the results, two authors (Lao X and Peng Q) entered the data into the statistical software programs independently with the same results.

## Result

### Identification of relevant studies

**Figure 1:**
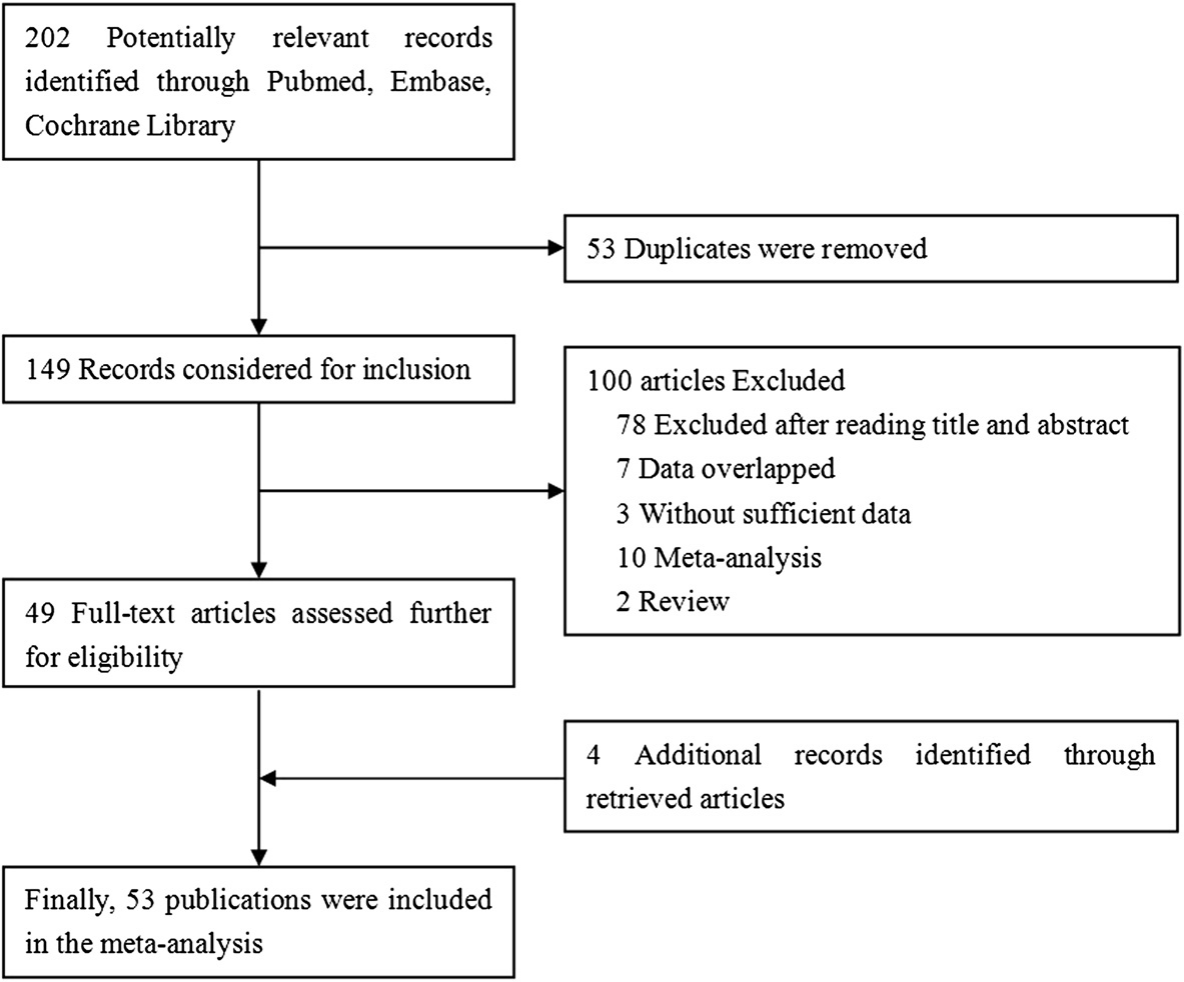
**Flowchart of the selection of studies for inclusion in the meta-analysis.**

After comprehensive searching, a total of 202 articles were retrieved, but only 49 full-text publications [15-21,23-27,29-65] which catered to the inclusion criteria were finally included in our meta-analysis. Additonal four studies [13,14,22,28] were identified by reviewing the bibliographies of the retrieved articles (Figure 1). Besides, because there was a study [29] containing two different ethnic populations (Caucasians and Negroids), we treat it as two individual case–control studies. Thus, in our meta-analysis we initially included a total of 54 studies which assessed the associations between GSTM1 polymorphism and gastric cancer. The 54 studies were published from 1991 to 2013 with 35 were carried out in Asian countries, 11 in Europe countries, and eight in America. Of these 54 studies, 51 were case–control design, while the other three were nested case–control design from cohort. The number of cases in the included studies for GSTM1 deletion varied from 5 to 1225 patients. There were 14 studies focused on the joint effect of GSTM1 null genotype and smoking status on gastric cancer risk, four investigated the joint effect of GSTM1 null genotype and alcohol drinking, and seven eveluated the joint effect of GSTM1 null genotype and *Helicobacter pylori* infection. 15 studies investigated the gene-gene interaction between GSTM1 and GSTT1 polymorphisms in the association with gastric cancer risk. Table 1 presents a brief description of these 54 studies.

**Table 1:** **Characteristics of included studies of gastric cancer and GSTM1 status**

**Study**	**Ethnicity (region)**	**No. of cases/controls**	**GC diagnosis**	**Source of control selection**	**Matching criteria**	**Genotyping method**	**Exposures**	**Null GSTM1 n(%)**	
								**Case**	**Control**
Richard C. Strange 1991 [13]	Caucasian (Britain)	19/49	Histologically confirmed	Clinic based	NA	Horizontal starch gel electrophoresis	NA	14(73.7)	20(40.8)
Shoji Harada 1992 [14]	Asian (Japan)	19/84	NA	Healthy volunteers	NA	PCR	NA	14(73.7)	40(47.6)
Shunji Kato 1996 [15]	Asian (Japan)	64/120	Histologically confirmed	Clinic based	Age,gender	PCR-RFLP	NA	30(46.9)	61(50.8)
Takahiko Katoh 1996 [16]	Asian (Japan)	139/126	Histologically confirmed	Healthy volunteers	Age	Multiplex PCR	Smoking status	79(56.8)	55(43.6)
Mark Deakin 1996 [17]	Caucasian (Britain)	136/577	NA	Clinic based	NA	PCR	NA	72(52.9)	316(54.8)
Liakhovich VV 1997 [18]	Caucasian (Russia)	49/53	NA	Healthy volunteers	NA	PCR	NA	21(42.9)	21(39.6)
Enders K. W. Ng 1998 [19]	Asian (China)	35/35	NA	Clinic based	Age,gender	Differential PCR	*Helicobacter pylori* infection	23(65.7)	13(52.0)
Gisela Martins 1998 [20]	Caucasian (Portugal)	148/84	Histologically diagnosis	Healthy volunteers	NA	Differential PCR	NA	71(48.0)	44(52.0)
Veronica Wendy Setiawan 2000 [21]	Asian (China)	87/419	Pathologically diagnosis	Population based	Geographical origin	PCR	Smoking status, alcohol drinking and *Helicobacter pylori* infection	42(48.3)	212(50.6)
Qing Lan 2001 [22]	Caucasian (Poland)	347/426	NA	Population based	Age,gender	PCR-RFLP	NA	167(48.1)	222(52.1)
Lin Cai 2001 [23]	Asian (China)	95/94	Histologically or operation diagnosis	Population based	Age,gender	PCR	Smoking status and GSTT1 genotyping	60(63.2)	43(45.7)
Iraj Saadat 2001 [24]	Caucasian (Iran)	42/131	Pathologically diagnosis	Healthy volunteers	Age,gender	PCR	GSTT1 genotyping	26(61.9)	53(40.5)
Alessandro Sgambato 2002 [25]	Caucasian (Italy)	8/100	NA	Healthy volunteers	NA	PCR	NA	5(62.5)	53(53.0)
Ming-Shiang Wu 2002 [26]	Asian (China)	356/278	Histologically diagnosis	Healthy volunteers	NA	Multiplex PCR	NA	173(48.6)	136(48.9)
Chang-Ming Gao 2002 [27]	Asian (China)	153/223	Histopathologically diagnosis	Population based	Age,gender	Multiplex PCR	Smoking status	90(58.8)	133(59.6)
Suck Chei Choi 2003 [28]	Asian (South Korea)	80/177	Pathologically diagnosis	Healthy volunteers	NA	PCR	NA	46(57.5)	95(53.7)
Jucimara Colombo1 2004 [29]	Caucasian (Brazil)	87/135	Histopathologically diagnosis	Healthy volunteers	Age,gender	Multiplex PCR	NA	45(51.7)	60(44.4)
Jucimara Colombo2 2004 [29]	Negroid (Brazil)	13/15	Histopathologically diagnosis	Healthy volunteers	Age,gender	Multiplex PCR	NA	2(15.4)	2(13.3)
Mark J. Roth 2004 [30]	Asian (China)	90/454	Pathologically, radiologically, cytological or operation diagnosis	Healthy cohort subjects	Age,gender	RT-PCR	NA	66(73.3)	145(31.9)
Shioto Suzuki 2004 [31]	Asian (Japan)	145/177	NA	Clinic based	Age	PCR	NA	87(60.0)	84(47.5)
Auxiliadora González 2004 [32]	Caucasian (Costa Rica)	31/51	Pathologically diagnosis	Healthy volunteers	NA	Multiplex PCR	NA	15(48.4)	26(51.0)
María M. Torres 2004 [33]	Caucasia (Colombia)	46/96	Pathologically diagnosis	Healthy volunteers	Age,gender	Multiplex PCR	NA	30(65.2)	36(37.5)
Jing Shen 2005 [34]	Asian (China)	112/675	Endoscopic and pathological diagnosis	Healthy volunteers	NA	PCR	Smoking status	71(63.4)	361(53.5)
Kuang-Chi Lai 2005 [35]	Asian (China)	123/121	Histologically or operation diagnosis	Healthy volunteers	NA	Multiplex PCR	NA	73(59.3)	55(45.5)
Hao Li 2005 [36]	Asian (China)	100/62	Pathologically diagnosis	Clinic based	NA	PCR	Smoking status and *Helicobacter pylori* infection	67(67.0)	26(41.9)
Li-Na Mu 2005 [37]	Asian (China)	196/393	Pathologically diagnosis	Population based	Age,gender	PCR	NA	127(64.8)	235(59.8)
Hong-Mei Nan 2005 [38]	Asian (South Korea)	400/614	Histologically diagnosis	Clinic based	Age,gender	Multiplex PCR	NA	251(62.8)	360(58.6)
Lulufer Tamer 2005 [39]	Caucasian (Turkey)	70/204	Histologically or operation diagnosis	Population based	NA	RT-PCR	Smoking status and GSTT1 genotyping	40(57.1)	88(43.1)
Antonio Agudo 2006 [40]	Caucasian (Britain)	242/932	Pathologically diagnosis	Healthy cohort subjects	Age, gender, center and date of blood collection	PCR	Smoking status	122(50.4)	498(53.4)
Kuen Lee 2006 [41]	Caucasian (Chile)	73/263	Histologically diagnosis	Clinic based	NA	PCR	Smoking status and alcohol drinking	13(17.8)	56(21.3)
Carmen Martinez 2006 [42]	Caucasian (Spain)	87/329	Histologically diagnosis	Healthy volunteers	Geographical origin	Multiplex PCR	GSTT1 genotyping	33(37.9)	149(45.3)
Su Hyung Hong 2006 [43]	Asian (South Korea)	108/238	Histologically diagnosis	Healthy volunteers	NA	Multiplex PCR	Smoking status and alcohol drinking and *Helicobacter pylori* infection	60(55.6)	134(56.3)
Stefania Boccia 2007 [44]	Caucasian (Italy)	107/254	Histologically diagnosis	Clinic based	Age,gender	Multiplex PCR	Smoking status and alcohol drinking	59(56.2)	135(52.7)
Annamaria Ruzzo 2007 [45]	Caucasian (Italy)	79/112	Pathologically diagnosis	Population based	Age,gender	Multiplex PCR	*Helicobacter pylori* infection and GSTT1 genotyping	35(44.3)	61(54.5)
Louise Wideroff 2007 [46]	Caucasian (America)	116/208	Histologically diagnosis	Population based	Age,gender	PCR	NA	61(52.6)	121(58.2)
Shweta Tripathi 2008 [47]	Caucasian (India)	76/100	Histopathologically diagnosis	Clinic based	Age,gender	PCR	GSTT1 genotyping	31(40.8)	39(39.0)
Mansour S. Al-Moundhri 2009 [48]	Caucasian (Oman)	107/107	NA	Healthy volunteers	Geographical origin	Multiplex PCR	*Helicobacter pylori* infection and GSTT1 genotyping	42(39.3)	32(30.0)
Mohammad Masoudi 2009 [49]	Caucasian (Iran)	67/134	Pathologically diagnosis	Healthy volunteers	Age,gender	PCR	NA	37(55.2)	60(44.8)
Manzoor A. Malik 2009 [50]	Caucasian (India)	108/195	Histopathologically diagnosis	Healthy volunteers	Age	Multiplex PCR	Smoking status	64(59.3)	79(40.5)
Kristin A. Moy 2009 [51]	Asian (China)	170/735	Histopathologically, clinically, radiologically or operation diagnosis	Healthy cohort subjects	Age and date of biospecimen collection	TaqMan	GSTT1 genotyping	98(57.6)	415(56.5)
Kazem Zendehdel 2009 [52]	Caucasian (Sweden)	124/469	NA	Population based	Age,gender	Multiplex PCR	Smoking status	70(56.5)	239(51.0)
Jin-Mei Piao 2009 [53]	Asian (South Korea)	2213/1699	Histologically diagnosis	Healthy volunteers	NA	TaqMan	GSTT1 genotyping	1225(55.4)	923(54.3)
Thai V. Nguyen 2010 [54]	Asian (Vietnam)	59/109	NA	Clinic based	NA	PCR	NA	43(73.0)	75(69.0)
Domenico Palli 2010 [55]	Caucasian (Italy)	296/546	Histologically diagnosis	Population based	NA	Multiplex PCR	GSTT1 genotyping	166(56.1)	275(50.4)
Dhirendra Singh Yadav 2010 [56]	Caucasian (India)	133/270	Histopathologically diagnosis	Healthy volunteers	Age,gender	Multiplex PCR	NA	49(37.0)	120(44.0)
Mohamad Darazy 2011 [57]	Caucasian (Lebanon)	13/70	Histologically diagnosis	Healthy volunteers	Age,gender	PCR	NA	6(46.2)	12(17.1)
Ya-ping Luo 2011 [58]	Asian (China)	123/129	Pathologically diagnosis	Healthy volunteers	NA	PCR	NA	93(75.6)	71(55.0)
An-Ping Zhang 2011 [59]	Asian (China)	194/412	Histologically diagnosis	Healthy volunteers	NA	PCR-CTPP	GSTT1 genotyping	105(54.1)	194(47.1)
Deepmala Yadav 2011 [60]	Caucasian (India)	41/130	Pathologically diagnosis	Healthy volunteers	Geographical origin	Multiplex PCR	GSTT1 genotyping	11(26.8)	38(29.2)
Mª Asunción García-González 2012 [61]	Caucasian (Spain)	557/557	Histopathologically diagnosis	Clinic based	Age,gender	Multiplex PCR	*Helicobacter pylori* infection and GSTT1 genotyping	283(50.8)	267(47.9)
Chen Jing 2012 [62]	Asian (China)	410/410	Histologically diagnosis	Healthy volunteers	Age,gender	PCR-CTPP	GSTT1 genotyping	240(58.6)	207(50.6)
Mridul Malakar 2012 [63]	Caucasian (India)	102/204	Histopathologically diagnosis	Population based	Age,gender	PCR	Smoking status and GSTT1 genotyping	57(55.9)	97(47.5)
Aptullah Haholu 2013 [64]	Caucasian (Turkey)	50/57	Pathologically diagnosis	Population based	Age,gender	Multiplex PCR	NA	26(52.0)	25(43.9)
Sang-Yong Eom 2013 [65]	Asian (South Korea)	477/476	Histologically diagnosis	Healthy volunteers	Age,gender	Multiplex PCR	NA	263(55.1)	259(54.4)

GC, gastric cancer; NA: relative data not available in original studies; PCR: Polymerase chain reaction; PCR–RFLP: Polymerase chain reaction-restriction fragment length polymorphism; RT-PCR: Real-Time PCR; PCR-CTPP: Polymerase chain reaction with confronting two -pair primers.

### Meta-analysis results

Table 2 lists the main results of this meta-analysis.

**Table 2:** **Summary of pooled odds ratios (OR) with confidence intervals (CI) of the GSTM1 polymorphism and gastric cancer risk**

**Group of analysis**	**n** ^**†**^	GSTM1 (Null vs. Present*)		**M** ^**#**^	**Heterogeneity**	
		**OR (95% CI)**	**P**_**OR**_		**I** ^**2**^ **(%)**	**P**_**Q**_^**※**^
Overall	54	1.207(1.106-1.317)	<0.001	R	49.9	<0.001
**Ethnicity**						
Asians	24	1.264(1.164-1.422)	<0.001	R	51.8	0.002
Caucasians	29	1.154(1.008-1.321)	0.037	R	50.6	0.001
Negroids	1	1.182(0.142-9.827)	0.887	F	—^¶^	—^¶^
**Smoking status**						
Non-smokers	14	1.370(1.043-1.800)	0.024	R	46.7	0.028
Ever-smokers	14	1.558(1.111-2.183)	0.010	R	69.6	<0.001
**Alcohol drinking**						
Non-drinkers	4	0.872(0.623-1.220)	0.425	F	0.0	0.757
Ever-drinkers	4	1.112(0.771-1.602)	0.570	F	0.0	0.905
***Helicobacter pylori*** **infection**						
*Helicobacter pylori* negative	7	0.869(0.654-1.156)	0.334	F	6.8	0.373
*Helicobacter pylori* positive	7	1.595(1.104-2.304)	0.013	R	49.9	0.076

†Number of studies included.

*The genetic comparison model for GSTM1–GSTT1interaction analysis is Dual null genotype vs. Non-null genotype.

# M, model of meta-analysis; R, random-effects model; F, fixed-effects model.

^※^ P_Q_: P values of Q-test for heterogeneity test.

^¶^Values could not be calculated out.

**Figure 2:**
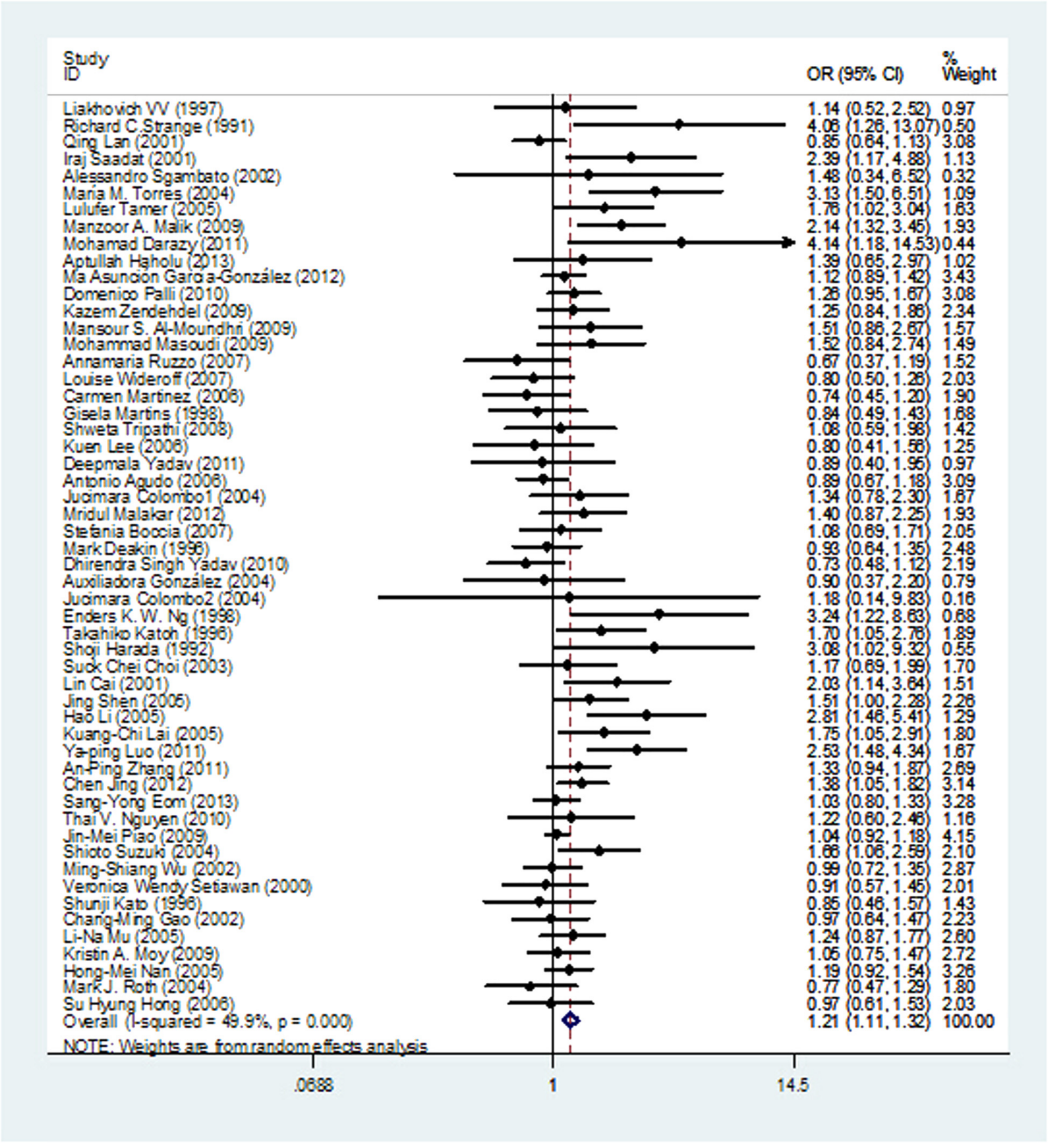
**Forest plots for the null genotype of GSTM1 and gastric cancer risk of overall populations using a random-effects model (null genotype vs. present genotype).**

The results of pooling all studies showed that the null genotype of GSTM1 was associated with an increased gastric cancer risk (OR = 1.207, 95% CI: 1.106-1.317, P < 0.001), using the random-effects model (I^2^ : 49.9%, P_Q_ < 0.001) (Figure 2). As shown in Tables 2, specific data were stratified, on the basis of ethnicity, into three subgroups: Aians, Caucasians and Negroids. Statistically significant findings were found in Asians and Caucasians but not in Negroids. The pooled OR were 1.264 (95% CI: 1.164-1.422, P < 0.001, P for heterogeneity = 0.002) in Aians, 1.154 (95% CI: 1.008-1.321, P < 0.037, P for heterogeneity = 0.001) in Caucasians, and 1.182 (95% CI: 0.142-9.827, P < 0.887) in Negroids, respectively.

The data were also stratified, in accordance with the smoking status, into non-smokers and ever-smokers subgroups. Statistically significant findings between the null genotype of GSTM1 and gastric cancer risk was found in both non-smokers (OR = 1.370, 95% CI: 1.043-1.800, P < 0.024, P for heterogeneity = 0.028) and ever-smokers subgroups (OR = 1.558, 95% CI: 1.111-2.183, P < 0.010, P for heterogeneity < 0.001), respectively. The data were additionally stratified, in line with alcohol driking, into the subgroup of non-drinkers and ever-drinkers. However, null results were noted in both non-drinkers (OR = 0.872, 95% CI: 0.623-1.220, P = 0.425, P for heterogeneity = 0.757) and ever-drinkers (OR = 1.112, 95% CI: 0.771-1.602, P = 0.570, P for heterogeneity = 0.905). The data were further stratified, in the light of *Helicobacter pylori* infection, into *Helicobacter pylori* negative and *Helicobacter pylori* positive subgroups. Statistically significant findings could be found only in *Helicobacter pylori* positive subgroup (OR = 1.595, 95% CI: 1.104-2.304, P < 0.013 P for heterogeneity = 0.076), but not for *Helicobacter pylori* negative subgroup (OR = 0.869, 95% CI: 0.654-1.156, P < 0.334, P for heterogeneity = 0.373).

Table 3 shows the OR and 95% CI of GSTM1 and GSTT1 combined genotypes in gastric cancer cases and controls from 15 studies. We designated the present genotype individuals for both GSTM1 and GSTT1 genes as reference groups. There was an interaction that only observed for individuals with combined deletion mutations of GSTT1 and GSTM1 genes for gastric cancer risk (OR = 1.505, 95% CI: 1.165-1.944, P = 002). This shows that the null genotype of GSTM1 might increase gastric cancer risk associated with the GSTT1 null genotype.

**Table 3:** **Combined genotype analysis of GSTM1 and GSTT1 on risk of gastric cancer**

**GSTM1 genotyping**	**GSTT1 genotyping**	**Cases (n = 5072)**	**Control (n = 5775)**	**OR (95% CI)**	**P**_**OR**_
Present	Present	1315	1762	1	
	Null	1024	1126	1.107(0.980-1.251)	0.102
Null	Present	1461	1736	1.134(0.969-1.328)	0.117
	Null	1272	1151	1.505(1.165-1.944)	0.002

### Sensitivity analysis and publication bias

A sensitivity analysis was performed by the sequential omission of individual studies. The significance of the pooled OR in both the overall analysis and subgroup analysis were not influenced excessively by omitting any single study (data were not shown).

**Figure 3:**
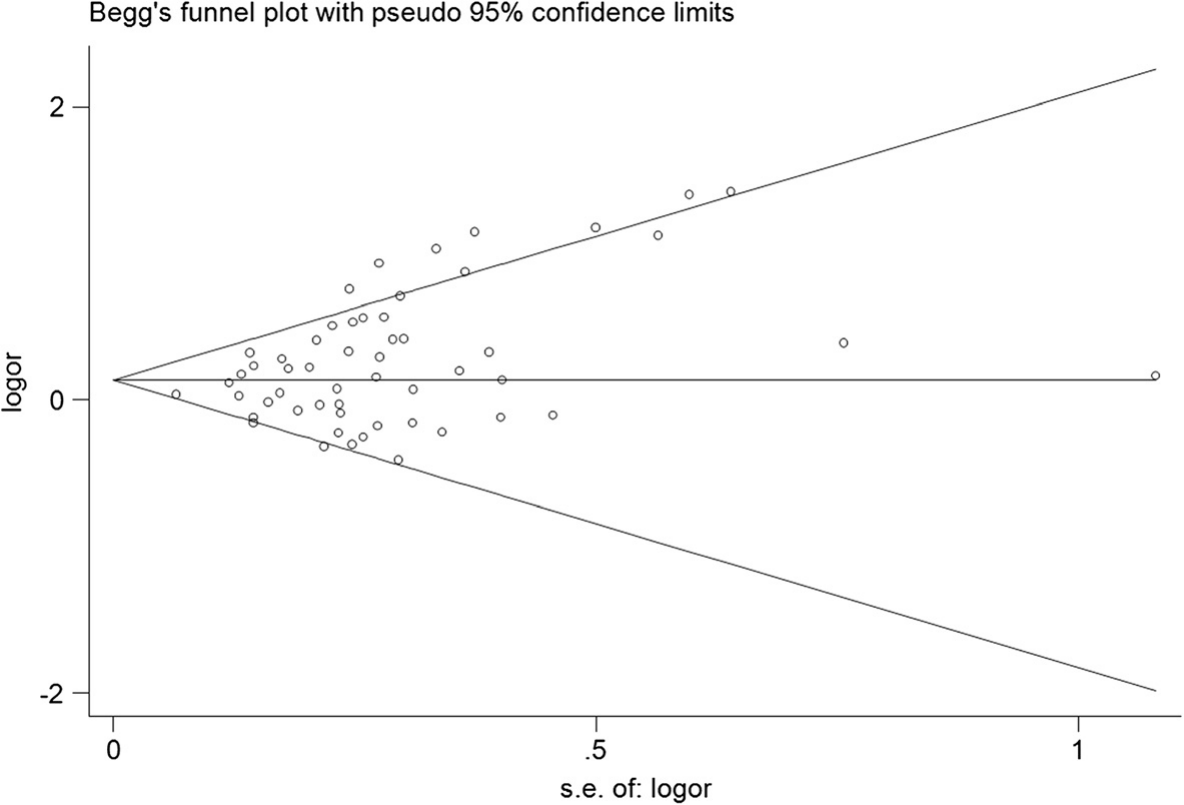
**Begg’s funnel plots for publication bias of the null genotype of GSTM1 and gastric cancer risk in the overall populations (null genotype vs. present genotype).** Each point represents a separate study for the indicated association.

Begg’s funnel plot and Egger’s test were performed to access the publication bias of literatures. As shown in Figure 3, the shape of the funnel plots seemed asymmetrical suggesting the presence of publication bias. Then, the Egger’s test was adopted to provide statistical evidence of funnel plot asymmetry. As expected, the results have shown that publication bias was evident in this meta-analysis (P = 0.004). Hence, the non-parametric “trim and fill” method [71], suggested by the Duval and Tweedie, was used to adjust for it. Meta-analysis with and without “trim and fill” method did not draw different conclusion (data not shown), indicating that our results were statistically robust.

## Discussion

The present and null genotype of GSTM1 is one of the most widely studied metabolic gene polymorphisms as susceptibility factor for gastric cancer. In 2005, La Torre et al. [72] firstly performed a meta-analysis about the association between GSTM1 genetic polymorphism and gastric cancer susceptibility, and the results suggested that GSTM1 deficiency possiblely has no effect on gastric cancer risk, but may modulate tobacco-related carcinogenesis of gastric cancer. Since then, additional expanding body of literatures assessing such association were conducted by Chen et al. [73] and Zhu et al. [74]. Both of them found that a excess gastric cancer risk was probably associated with GSTM1 null genotype in Asians, but not in Caucasians. Besides, Chen et al. revealed that smoking, *Helicobacter pylori* infection status did not modify the association between GSTM1 null genotype and gastric cancer risk. However, these previous meta-analysis did not cover all eligible studies published in Pubmed and even contain the overlapped data, which made their conclusions doubtable. What’s more, 12 new case–control studies [54-65] have been published since 2010. Hence, to derive the most comprehensive assessment of the associations between the GSTM1 polymorphism and gastric cancer risk, we undertook an updated meta-analysis of all available studies. The main finding of this meta-analysis of 54 studies involving 9,322 cases and 15,118 controls is that individuals of GSTM1 null genotype appear to have a significant increased risk of gastric cancer.

Large ethnic differences for the frequency of homozygous deletion in GSTM1 have been observed. Frequencies of homozygous GSTM1 null genotype in Japanese, Chinese, Indians (Asia), Caucasians and Africans were 48%-51%, 35%-63%, 33%-36%, 50%, 22%-35%, respectively [75]. For the subgroup meta-analyses of ethnicity, which categorized as Asians, Caucasians and Negroids, the results showed that the GSTM1 null phenotype predisposes to gastric cancer in both Asian and Caucasian populations, but not in Negroids. For Caucasians, what is notable that the association between GSTM1 null status and gastric cancer risk simply reaches a slightly statistically significant level. Since the Caucasian reports in the subgroup analysis include a mixture of populations from very distant countries, the result must be interpreted with caution. For Negroids, it might seems that the low prevalence of this viriant and the low number of samples would make it difficult to detect an association between GSTM1 null phenotype and gastric cancer risk with only 13 cases and 15 controls. It may also be that other gastric cancer risk factors, such as prevalence of *Helicobacter pylori* infection, lifestyle, diet, or other environmental risk factors for which ethnic groups vary, may be responsible for the different associations observed in this meta-analysis.

In our meta-analysis, we proceeded the subgroup meta-analysis of pooling data from the cases and the controls on smoking status, and the suggestion of an increase risk of gastric cancer was found in not only non-smokers but also ever-smokers. Interestingly, both the OR and the upper and lower limits of corresponding 95% CI in ever-smokers are much higher than non-smokers. Smoking is an important established risk factor for gastric cancer that accouts for about 50 % increase in gastric cancer risk [76]. Jarebinski et al. [77], in 1992 first reported that there is a weak association between smoking and gastric cancer. Then, Ladeiras-Lopes et al. [78] in 2008 conducted a meta-analysis including only prospective studies to estimate the relationship between smoking and gastric cancer, and concluded that smoking is the most important behavioral risk factor for gastric cancer, especially for male smokers. Nevertheless, the mechanism by which tobacco smoke facilitates gastric cancer development is not well recognised. Processed tobacco contains over 3,000 compounds including 30 carcinogens. The mainstream and sidestream smoke generated when tobacco in cigarettes is burnt contains more than 4,000 constituents including about 50 carcinogens (e.g. benzo(a)pyrene, styrene-7,8-oxide, transstibene oxide, epoxybutanes, ethylene oxide, halomethanes, and methyl bromide). Many of these compounds are firstly metabolized by phase I enzymes, and detoxified and converted to inactive metabolites by the phase II enzymes including GST family enzymes. Individuals who present inefficient phase II metabolism may accumulate more toxic intermediates when exposed to smoking, and thereby resulting in increasing their risk of developing cancers. GSTM1 is one of the major member of the GST family which are involved in detoxification of smoking-related carcinogens. Previous reports have showed that the null genotype of GSTM1 causes the loss of enzymic activity [14]. So, we propose a hypothesis that smokers who present the GSTM1 null genotype are more susceptible to impairment by tobacco smoke, due to the low catalytic efficiency.

Furthermore, we carried out the subgroup analysis on alcohol drinking to detect the possible effect of GSTM1 deficiency on gastric cancer risk. Alcohol drinking is supposed to be a risk factor of gastric cancer, due to the reactive oxygen species (ROSs). ROSs are produced during alcohol metabolism as a result of the generation of NADH from the conversion of ethanol to acetaldehyde by alcohol dehydrogenase, and may induce gastric mucosal oxidative injury [79,80]. Despite the biological plausibility of alcohol drinking as a modulator of gastric cancer susceptibility, previously inconsistent results have appeared. In 1988, an International Agency for Research on Cancer (IARC) working group concluded that there was no evidence support that alcohol dringking was involved in the pathogenesis of gastric cancer [81]. In 2007, the IARC working group reassessed the carcinogenesis of alcohol consumption and concluded that alcohol consumption might be associated with an increased gastric cancer risk, but confounding by smoking and dietary habits could not be ruled out [82]. GSTM1 can detoxify many toxicological substrates, including ethanol, to become inactive products. Therefore, the effect of the interactions between GSTM1 polymorphism and alcohol drinking on gastric cancer risk should be noted. Unfortunately, there was no association between GSTM1 null genotype and increased gastric cancer risk was found in non-drinkers or ever drinkers in the meta-analysis. However, only four studies with insufficient statistic power concerning drinking status were included in the present mata-analysis, with possible biases existed. Further investigations on the effect of the interactions of GSTM1 null genotpe and alcohol drinking on gastric cancer risk are required to address this controversy.

We also examined the association between *Helicobacter pylori* infection and the risk of gastric cancer. *Helicobacter pylori* is a spiral Gram-negative bacterium that colonizes the stomach, and which has been implicated as a Class 1 human carcinogen for gastric cancer [83]. The promulgation was based on several scrupulously conducted case–control studies in which chronic *Helicobacter pylori* infection was shown to eveluate the risk of gastric cancer from 2.8-to 6-fold [84,85]. There is now evidence that approximately 25–50% of the world’s population is infected by the microorganism, and that up to 85 % of noncardia gastric cancers are *Helicobacter pylori* related [86]. However, it remains obscure why quite a few individuals infected do not develop the malignancy, even in areas with a high prevalence of both *Helicobacter pylori* infection and gastric cancer. Ng EK et al. found that GSTM1 null genotype was more prevalent in gastric cancer cases with *Helicobacter pylori* infection than without the infection, and the result suggested that the absence of GST-mu function might have enhanced the susceptibility of these patients to the carcinogenic process initiated or facilitated by *Helicobacter pylori* [19]. In our meta-analysis, there were six publications including a total of 1,007 cases and 1,487 controls examined the relationship between *Helicobacter pylori* infection and GSTM1 polymorphism for the risk of gastric cancer, and some risk excess was observed among *Helicobacter pylori* infection positive individuals compared with negative individuals by combining the data available from these six studies. However, these results should be interpreted with caution as the subset analysis involved a small number of patients and controls that may have affected the statistical analysis.

If genetic susceptibility to gastric cancer is, in part, mediated through metabolic gene polymorphisms, it is possible that the combinations of certain genotypes may be more discriminating as risk factors for gastric cancer than a single locus genotype. Because GSTM1 and GSTT1 are involved in the detoxification of a variety of compounds, and their substrates often overlap, it is possible that individuals with a more defective genotype of these genes can be expected to at higher risk of cancers. A previous study has looked at the combination between GSTM1 and GSTT1 in gastric cancer and showed that a 95% significant increased risk of gastric cancer appeared for individuals with combined deletion mutations in GSTT1 and GSTM1 genes in comparison with individuals with both homozygous wild genotypes [87]. Hence, we also investigate the possible interaction between GSTT1 and GSTM1 status and gastric cancer risk in this meta-analysis. Even though only 15 of 54 selected studies collected data on GSTT1 status, a statistically significant increased risk for gastric cancer was detected for individuals with combined deletion mutations in both GSTM1 and GSTT1 genes compared to those with wild genotypes by pooling the data from available studies.

Several limitations of the study have to be acknowledged in interpreting the results. Firstly, the sample sizes for a majority of included studies were relatively small; source of controls were somewhat different from each other; the matching criteria for the cases and controls were also not strict. Thus, robust guarantee could hardly be made among all those eligible studies. Additionally, only published studies were included in our meta-analysis and a publication bias have occurred. Even though non-parametric “trim and fill” method was used to adjust for it and the result suggested that meta-analysis with and without “trim and fill” method did not draw different conclusion, if the unpublished studies are not included an overestimation of the GSTM1 null effect may inevitably appear. Finally, the subgroup meta-analyses considering interactions between GSTM1 null genotype and smoking status, alcohol drinking and *Helicobacter pylori* infection, as well as GSTT1 null genotype were performed by collecting data from a minority of included studies, so selection bias may have occurred in this meta-analysis. What’s more, more subgroup analyses performed on age, gender, histological types, and other factors (such as vegetable and fruit intakes, salt and salted preserved food intakes) would be better to investigate possible biases; however, we could not perform subgroup analyses on these factors owing to the limited available information in the primary literatur.

## Conclusion

This meta-analysis suggests that the null genotype of GSTM1 are associated with increased gastric cancer risk, and the subgroup meta-analysis on the basis of ethnicity showed that significant associations are found for Asians and Caucasians, but not for Negroids. In additon, the null genotype of GSTM1 may modulate the smoking-related and *Helicobacter pylori*-related carcinogenesis of gastric cancer, and that the combination of unfavourable GSTT1 polymorphism may result in an additional risk of gastric cancer. Future studies with large and carefully design are warranted to better understand such a association between GSTM1 null genotype and gastric cancer risk.

## References

[1] Bertuccio P, Chatenoud L, Levi F, Praud D, Ferlay J, Negri E, Malvezzi M, La Vecchia C (2009). Recent patterns in gastric cancer: a global overview. Int J Cancer.

[2] Jemal A, Bray F, Center MM, Ferlay J, Ward E, Forman D (2011). Global cancer statistics. CA Cancer J Clin.

[3] Parkin DM, Pisani P, Ferlay J (1993). Estimates of the worldwide incidence of eighteen major cancers in 1985. Int J Cancer.

[4] Neugut AI, Hayek M, Howe G (1996). Epidemiology of gastric cancer. Semin Oncol.

[5] Zaridze D, Borisova E, Maximovitch D, Chkhikvadze V (2000). Alcohol consumption, smoking and risk of gastric cancer: case–control study from Moscow. Russia Cancer Causes Control.

[6] Stadtlander CT, Waterbor JW (1999). Molecular epidemiology, pathogenesis and prevention of gastric cancer. Carcinogenesis.

[7] Perera FP, Weinstein IB (2000). Molecular epidemiology: recent advances and future directions. Carcinogenesis.

[8] Ragin CC, Langevin S, Rubin S, Taioli E (2010). Review of studies on metabolic genes and cancer in populations of African descent. Genet Med.

[9] Hayes JD, Pulford DJ (1995). The glutathione S-transferase supergene family: regulation of GST and the contribution of the isoenzymes to cancer chemoprotection and drug resistance. Crit Rev Biochem Mol Biol.

[10] Board P, Coggan M, Johnston P, Ross V, Suzuki T, Webb G (1990). Genetic heterogeneity of the human glutathione transferases: a complex of gene families. Pharmacol Ther.

[11] Wang Q, Chen Y, Zhang Y, Xu W, He H, Li X, Cui H (2014). Quantitative assessment of the influence of glutathione S-transferase T1 null variant on gastric cancer risk. Tumour Biol.

[12] Gonzalez CA, Sala N, Capella G (2002). Genetic susceptibility and gastric cancer risk. Int J Cancer.

[13] Strange RC, Matharoo B, Faulder GC, Jones P, Cotton W, Elder JB, Deakin M (1991). The human glutathione S-transferases: a case–control study of the incidence of the GST1 0 phenotype in patients with adenocarcinoma. Carcinogenesis.

[14] Harada S, Misawa S, Nakamura T, Tanaka N, Ueno E, Nozoe M (1992). Detection of GST1 gene deletion by the polymerase chain reaction and its possible correlation with stomach cancer in Japanese. Hum Genet.

[15] Kato S, Onda M, Matsukura N, Tokunaga A, Matsuda N, Yamashita K, Shields PG (1996). Genetic polymorphisms of the cancer related gene and Helicobacter pylori infection in Japanese gastric cancer patients. An age and gender matched case–control study. Cancer.

[16] Katoh T, Nagata N, Kuroda Y, Itoh H, Kawahara A, Kuroki N, Ookuma R, Bell DA (1996). Glutathione S-transferase M1 (GSTM1) and T1 (GSTT1) genetic polymorphism and susceptibility to gastric and colorectal adenocarcinoma. Carcinogenesis.

[17] Deakin M, Elder J, Hendrickse C, Peckham D, Baldwin D, Pantin C, Wild N, Leopard P, Bell DA, Jones P, Deakin M, Elder J, Hendrickse C, Peckham D, Baldwin D, Pantin C, Wild N, Leopard P, Bell DA, Jones P, Duncan H, Brannigan K, Alldersea J, Fryer AA, Strange RC (1996). Glutathione S-transferase GSTT1 genotypes and susceptibility to cancer: studies of interactions with GSTM1 in lung, oral, gastric and colorectal cancers. Carcinogenesis.

[18] Liakhovich VV, Vavilin VA, Gutkina NI, Laktionova IP, Makarova SI, Mitrofanov DV, Ostashevskii VA, Chasovnikova OB (1997). Genes and enzymes of the xenobiotic-metabolizing system in cancer pathology. Vopr Med Khim.

[19] Ng EK, Sung JJ, Ling TK, Ip SM, Lau JY, Chan AC, Liew CT, Chung SC (1998). Helicobacter pylori and the null genotype of glutathione-S-transferase-mu in patients with gastric adenocarcinoma. Cancer.

[20] Alves GM (1998). Glutathione S transferase mu polymorphism and gastric cancer in the Portuguese population. Biomarkers.

[21] Setiawan VW, Zhang ZF, Yu GP, Li YL, Lu ML, Tsai CJ, Cordova D, Wang MR, Guo CH, Yu SZ, Kurtz RC (2000). GSTT1 and GSTM1 null genotypes and the risk of gastric cancer: a case–control study in a Chinese population. Cancer Epidemiol Biomarkers Prev.

[22] Lan Q, Chow WH, Lissowska J, Hein DW, Buetow K, Engel LS, Ji B, Zatonski W, Rothman N (2001). Glutathione S-transferase genotypes and stomach cancer in a population-based case–control study in Warsaw. Poland Pharmacogenetics.

[23] Cai L, Yu SZ, Zhang ZF (2001). Glutathione S-transferases M1, T1 genotypes and the risk of gastric cancer: a case–control study. World J Gastroenterol.

[24] Saadat I, Saadat M (2001). Glutathione S-transferase M1 and T1 null genotypes and the risk of gastric and colorectal cancers. Cancer Lett.

[25] Sgambato A, Campisi B, Zupa A, Bochicchio A, Romano G, Tartarone A, Galasso R, Traficante A, Cittadini A (2002). Glutathione S-transferase (GST) polymorphisms as risk factors for cancer in a highly homogeneous population from southern Italy. Anticancer Res.

[26] Wu MS, Chen CJ, Lin MT, Wang HP, Shun CT, Sheu JC, Lin JT (2002). Genetic polymorphisms of cytochrome p450 2E1, glutathione S-transferase M1 and T1, and susceptibility to gastric carcinoma in Taiwan. Int J Colorectal Dis.

[27] Gao CM, Takezaki T, Wu JZ, Li ZY, Liu YT, Li SP, Ding JH, Su P, Hu X, Xu TL, Sugimura H, Tajima K (2002). Glutathione-S-transferases M1 (GSTM1) and GSTT1 genotype, smoking, consumption of alcohol and tea and risk of esophageal and stomach cancers: a case–control study of a high-incidence area in Jiangsu Province. China Cancer Lett.

[28] Choi SC, Yun KJ, Kim TH, Kim HJ, Park SG, Oh GJ, Chae SC, Oh GJ, Nah YH, Kim JJ, Chung HT (2003). Prognostic potential of glutathione S-transferase M1 and T1 null genotypes for gastric cancer progression. Cancer Lett.

[29] Colombo J, Rossit AR, Caetano A, Borim AA, Wornrath D, Silva AE (2004). GSTT1, GSTM1 and CYP2E1 genetic polymorphisms in gastric cancer and chronic gastritis in a Brazilian population. World J Gastroenterol.

[30] Roth MJ, Abnet CC, Johnson LL, Mark SD, Dong ZW, Taylor PR, Dawsey SM, Qiao YL (2004). Polymorphic variation of Cyp1A1 is associated with the risk of gastric cardia cancer: a prospective case-cohort study of cytochrome P-450 1A1 and GST enzymes. Cancer Causes Control.

[31] Suzuki S, Muroishi Y, Nakanishi I, Oda Y (2004). Relationship between genetic polymorphisms of drug-metabolizing enzymes (CYP1A1, CYP2E1, GSTM1, and NAT2), drinking habits, histological subtypes, and p53 gene point mutations in Japanese patients with gastric cancer. J Gastroenterol.

[32] Gonzalez A, Ramirez V, Cuenca P, Sierra R (2004). Polymorphisms in detoxification genes CYP1A1, CYP2E1, GSTT1 and GSTM1 in gastric cancer susceptibility. Rev Biol Trop.

[33] Torres MM, Acosta CP, Sicard DM (2004). Groot de Restrepo H: Genetic susceptibility and risk of gastric cancer in a human population of Cauca. Colombia. Biomedica.

[34] Shen J, Wang RT, Xu YC, Wang LW, Wang XR (2005). Interaction models of CYP1A1, GSTM1 polymorphisms and tobacco smoking in intestinal gastric cancer. World J Gastroenterol.

[35] Lai KC, Chen WC, Tsai FJ, Li SY, Chou MC, Jeng LB (2005). Glutathione S-transferase M1 gene null genotype and gastric cancer risk in Taiwan. Hepatogastroenterology.

[36] Li H, Chen XL, Li HQ (2005). Polymorphism of CYPIA1 and GSTM1 genes associated with susceptibility of gastric cancer in Shandong Province of China. World J Gastroenterol.

[37] Mu LN, Lu QY, Yu SZ, Jiang QW, Cao W, You NC, Setiawan VW, Zhou XF, Ding BG, Mu LN, Lu QY, Yu SZ, Jiang QW, Cao W, You NC, Setiawan VW, Zhou XF, Ding BG, Wang RH, Zhao J, Cai L, Rao JY, Heber D, Zhang ZF (2005). Green tea drinking and multigenetic index on the risk of stomach cancer in a Chinese population. Int J Cancer.

[38] Nan HM, Park JW, Song YJ, Yun HY, Park JS, Hyun T, Youn SJ, Kim YD, Kang JW, Kim H (2005). Kimchi and soybean pastes are risk factors of gastric cancer. World J Gastroenterol.

[39] Tamer L, Ates NA, Ates C, Ercan B, Elipek T, Yildirim H, Camdeviren H, Atik U, Aydin S (2005). Glutathione S-transferase M1, T1 and P1 genetic polymorphisms, cigarette smoking and gastric cancer risk. Cell Biochem Funct.

[40] Agudo A, Sala N, Pera G, Capella G, Berenguer A, Garcia N, Palli D, Boeing H, Del Giudice G, Saieva C, Carneiro F, Berrino F, Sacerdote C, Tumino R, Panico S, Berglund G, Simán H, Stenling R, Hallmans G, Martínez C, Amiano P, Barricarte A, Navarro C, Quirós JR, Allen N, Key T, Bingham S, Khaw KT, Linseisen J, Nagel G (2006). No association between polymorphisms in CYP2E1, GSTM1, NAT1, NAT2 and the risk of gastric adenocarcinoma in the European prospective investigation into cancer and nutrition. Cancer Epidemiol Biomarkers Prev.

[41] Lee K, Caceres D, Varela N, Csendes DA, Rios RH, Quinones SL (2006). Allelic variants of cytochrome P4501A1 (CYP1A1), glutathione S transferase M1 (GSTM1) polymorphisms and their association with smoking and alcohol consumption as gastric cancer susceptibility biomarkers. Rev Med Chil.

[42] Martinez C, Martin F, Fernandez JM, Garcia-Martin E, Sastre J, Diaz-Rubio M, Agundez JA, Ladero JM (2006). Glutathione S-transferases mu 1, theta 1, pi 1, alpha 1 and mu 3 genetic polymorphisms and the risk of colorectal and gastric cancers in humans. Pharmacogenomics.

[43] Hong SH, Kim JW, Kim HG, Park IK, Ryoo JW, Lee CH, Sohn YK, Lee JY (2006). Glutathione S-transferases (GSTM1, GSTT1 and GSTP1) and N-acetyltransferase 2 polymorphisms and the risk of gastric cancer. J Prev Med Public Health.

[44] Boccia S, Sayed-Tabatabaei FA, Persiani R, Gianfagna F, Rausei S, Arzani D, La Greca A, D’Ugo D, La Torre G, van Duijn CM, Ricciardi G (2007). Polymorphisms in metabolic genes, their combination and interaction with tobacco smoke and alcohol consumption and risk of gastric cancer: a case–control study in an Italian population. BMC Cancer.

[45] Ruzzo A, Canestrari E, Maltese P, Pizzagalli F, Graziano F, Santini D, Catalano V, Ficarelli R, Mari D, Bisonni R, Giordani P, Giustini L, Lippe P, Silva R, Mattioli R, Torresi U, Latini L, Magnani M (2007). Polymorphisms in genes involved in DNA repair and metabolism of xenobiotics in individual susceptibility to sporadic diffuse gastric cancer. *Clin*. Clin Chem Lab Med.

[46] Wideroff L, Vaughan TL, Farin FM, Gammon MD, Risch H, Stanford JL, Chow WH (2007). GST, NAT1, CYP1A1 polymorphisms and risk of esophageal and gastric adenocarcinomas. Cancer Detect Prev.

[47] Tripathi S, Ghoshal U, Ghoshal UC, Mittal B, Krishnani N, Chourasia D, Agarwal AK, Singh K (2008). Gastric carcinogenesis: possible role of polymorphisms of GSTM1, GSTT1, and GSTP1 genes. Scand J Gastroenterol.

[48] Al-Moundhri MS, Alkindy M, Al-Nabhani M, Al-Bahrani B, Burney IA, Al-Habsi H, Ganguly SS, Tanira M (2009). Combined polymorphism analysis of glutathione S-transferase M1/G1 and interleukin-1B (IL-1B)/interleukin 1-receptor antagonist (IL-1RN) and gastric cancer risk in an Omani Arab Population. J Clin Gastroenterol.

[49] Masoudi M, Saadat I, Omidvari S, Saadat M (2009). Genetic polymorphisms of GSTO2, GSTM1, and GSTT1 and risk of gastric cancer. Mol Biol Rep.

[50] Malik MA, Upadhyay R, Mittal RD, Zargar SA, Modi DR, Mittal B (2009). Role of xenobiotic-metabolizing enzyme gene polymorphisms and interactions with environmental factors in susceptibility to gastric cancer in Kashmir Valley. J Gastrointest Cancer.

[51] Moy KA, Yuan JM, Chung FL, Wang XL, Van Den Berg D, Wang R, Gao YT, Yu MC (2009). Isothiocyanates, glutathione S-transferase M1 and T1 polymorphisms and gastric cancer risk: a prospective study of men in Shanghai. China Int J Cancer.

[52] Zendehdel K, Bahmanyar S, McCarthy S, Nyren O, Andersson B, Ye W (2009). Genetic polymorphisms of glutathione S-transferase genes GSTP1, GSTM1, and GSTT1 and risk of esophageal and gastric cardia cancers. Cancer Causes Control.

[53] Piao JM, Shin MH, Kweon SS, Kim HN, Choi JS, Bae WK, Shim HJ, Kim HR, Park YK, Choi YD, Kim SH (2009). Glutathione-S-transferase (GSTM1, GSTT1) and the risk of gastrointestinal cancer in a Korean population. World J Gastroenterol.

[54] Nguyen TV, Janssen MJ, van Oijen MG, Bergevoet SM, te Morsche RH, van Asten H, Laheij RJ, Peters WH, Jansent JB (2010). Genetic polymorphisms in GSTA1, GSTP1, GSTT1, and GSTM1 and gastric cancer risk in a Vietnamese population. Oncol Res.

[55] Palli D, Polidoro S, D’Errico M, Saieva C, Guarrera S, Calcagnile AS, Sera F, Allione A, Gemma S, Zanna I, Filomena A, Testai E, Caini S, Moretti R, Gomez-Miguel MJ, Nesi G, Luzzi I, Ottini L, Masala G, Matullo G, Dogliotti E (2010). Polymorphic DNA repair and metabolic genes: a multigenic study on gastric cancer. Mutagenesis.

[56] Yadav DS, Devi TR, Ihsan R, Mishra AK, Kaushal M, Chauhan PS, Bagadi SA, Sharma J, Zamoawia E, Verma Y, Nandkumar A, Saxena S, Kapur S (2010). Polymorphisms of glutathione-S-transferase genes and the risk of aerodigestive tract cancers in the Northeast Indian population. Genet Test Mol Biomarkers.

[57] Darazy M, Balbaa M, Mugharbil A, Saeed H, Sidani H, Abdel-Razzak Z (2011). CYP1A1, CYP2E1, and GSTM1 gene polymorphisms and susceptibility to colorectal and gastric cancer among Lebanese. Genet Test Mol Biomarkers.

[58] Luo YP, Chen HC, Khan MA, Chen FZ, Wan XX, Tan B, Ou-Yang FD, Zhang DZ (2011). Genetic polymorphisms of metabolic enzymes-CYP1A1, CYP2D6, GSTM1, and GSTT1, and gastric carcinoma susceptibility. Tumour Biol.

[59] Zhang AP, Liu BH, Wang L, Gao Y, Li F, Sun SX (2011). Glutathione S-transferase gene polymorphisms and risk of gastric cancer in a Chinese population. Asian Pac J Cancer Prev.

[60] Yadav D, Chandra R, Saxena R, Agarwal D, Agarwal M, Ghosh T, Agrawal D (2011). Glutathione-S-transferase M1 and T1 genes and gastric cancer: a case control study in North Indian population. Gene.

[61] Garcia-Gonzalez MA, Quintero E, Bujanda L, Nicolas D, Benito R, Strunk M, Santolaria S, Sopena F, Badia M, Hijona E, Pérez-Aísa MA, Méndez-Sánchez IM, Thomson C, Carrera P, Piazuelo E, Jiménez P, Espinel J, Campo R, Manzano M, Geijo F, Pellisé M, González-Huix F, Espinós J, Titó L, Zaballa M, Pazo R, Lanas A (2012). Relevance of GSTM1, GSTT1, and GSTP1 gene polymorphisms to gastric cancer susceptibility and phenotype. Mutagenesis.

[62] Jing C, Huang ZJ, Duan YQ, Wang PH, Zhang R, Luo KS, Xiao XR (2012). Glulathione-S-transferases gene polymorphism in prediction of gastric cancer risk by smoking and Helicobacter pylori infection status. Asian Pac J Cancer Prev.

[63] Malakar M, Devi KR, Phukan RK, Kaur T, Deka M, Puia L, Barua D, Mahanta J, Narain K (2012). Genetic polymorphism of glutathione S-transferases M1 and T1, tobacco habits and risk of stomach cancer in Mizoram. India Asian Pac J Cancer Prev.

[64] Haholu A, Berber U, Karagoz B, Tuncel T, Bilgi O, Demirel D (2013). Is there any association of glutathione S-transferase T1 (GSTT1) and glutathione S-transferase M1 (GSTM1) gene polymorphism with gastric cancers?. Pol J Pathol.

[65] Eom SY, Yim DH, Zhang Y, Yun JK, Moon SI, Yun HY, Song YJ, Youn SJ, Hyun T, Park JS, Kim BS, Lee JY, Kim YD, Kim H (2013). Dietary aflatoxin B1 intake, genetic polymorphisms of CYP1A2, CYP2E1, EPHX1, GSTM1, and GSTT1, and gastric cancer risk in Korean. Cancer Causes Control.

[66] DerSimonian R, Laird N (1986). Meta-analysis in clinical trials. Control Clin Trials.

[67] Higgins JP, Thompson SG (2002). Quantifying heterogeneity in a meta-analysis. Stat Med.

[68] Mantel N, Haenszel W (1959). Statistical aspects of the analysis of data from retrospective studies of disease. J Natl Cancer Inst.

[69] Botto LD, Khoury MJ (2001). Commentary: facing the challenge of gene-environment interaction: the two-by-four table and beyond. Am J Epidemiol.

[70] Egger M, Davey Smith G, Schneider M, Minder C (1997). Bias in meta-analysis detected by a simple, graphical test. BMJ.

[71] Duval S, Tweedie R (2000). Trim and fill: a simple funnel-plot-based method of testing and adjusting for publication bias in meta-analysis. Biometrics.

[72] La Torre G, Boccia S, Ricciardi G (2005). Glutathione S-transferase M1 status and gastric cancer risk: a meta-analysis. Cancer Lett.

[73] Chen B, Zhou Y, Yang P, Wu XT (2010). Glutathione S-transferase M1 gene polymorphism and gastric cancer risk: an updated analysis. Arch Med Res.

[74] Zhu Y, He Q, Wang J, Pan HF (2012). The association between GSTM1 polymorphism and gastric cancer risk: a meta-analysis. Mol Biol Rep.

[75] Rebbeck TR (1997). Molecular epidemiology of the human glutathione S-transferase genotypes GSTM1 and GSTT1 in cancer susceptibility. Cancer Epidemiol Biomarkers Prev.

[76] La Torre G, Chiaradia G, Gianfagna F, De Lauretis A, Boccia S, Mannocci A, Ricciardi W (2009). Smoking status and gastric cancer risk: an updated meta-analysis of case–control studies published in the past ten years. Tumori.

[77] Jarebinski M, Adanja B, Vlajinac H, Pekmezovic T, Sipetic S (1992). Evaluation of the association of cancer of the esophagus, stomach and colon with habits of patients. Vojnosanit Pregl.

[78] Ladeiras-Lopes R, Pereira AK, Nogueira A, Pinheiro-Torres T, Pinto I, Santos-Pereira R, Lunet N (2008). Smoking and gastric cancer: systematic review and meta-analysis of cohort studies. Cancer Causes Control.

[79] Brind AM, Hurlstone A, Edrisinghe D, Gilmore I, Fisher N, Pirmohamed M, Fryer AA (2004). The role of polymorphisms of glutathione S-transferases GSTM1, M3, P1, T1 and A1 in susceptibility to alcoholic liver disease. Alcohol Alcohol.

[80] Bhattacharyya A, Chattopadhyay R, Mitra S, Crowe SE (2014). Oxidative stress: an essential factor in the pathogenesis of gastrointestinal mucosal diseases. Physiol Rev.

[81] ᅟ**Alcohol drinking. IARC Working Group, Lyon, 13–20 October 1987.** *IARC Monogr Eval Carcinog Risks Hum* 1988, **44:**1–378. PMC6421508 3236394

[82] Baan R, Straif K, Grosse Y, Secretan B, El Ghissassi F, Bouvard V, Altieri A, Cogliano V (2007). Carcinogenicity of alcoholic beverages. Lancet Oncol.

[83] Uemura N, Okamoto S, Yamamoto S, Matsumura N, Yamaguchi S, Yamakido M, Taniyama K, Sasaki N, Schlemper RJ (2001). Helicobacter pylori infection and the development of gastric cancer. N Engl J Med.

[84] Forman D, Newell DG, Fullerton F, Yarnell JW, Stacey AR, Wald N, Sitas F (1991). Association between infection with Helicobacter pylori and risk of gastric cancer: evidence from a prospective investigation. BMJ.

[85] Nomura A, Stemmermann GN, Chyou PH, Kato I, Perez-Perez GI, Blaser MJ (1991). Helicobacter pylori infection and gastric carcinoma among Japanese Americans in Hawaii. N Engl J Med.

[86] Brenner H, Arndt V, Stegmaier C, Ziegler H, Rothenbacher D (2004). Is Helicobacter pylori infection a necessary condition for noncardia gastric cancer?. Am J Epidemiol.

[87] Boccia S, La Torre G, Gianfagna F, Mannocci A, Ricciardi G (2006). Glutathione S-transferase T1 status and gastric cancer risk: a meta-analysis of the literature. Mutagenesis.

